# Synergistic antifungal activity and mechanisms of dehydronuciferine combined with fluconazole against drug-resistant *Candida albicans*

**DOI:** 10.1128/spectrum.01619-26

**Published:** 2026-09-08

**Authors:** Xiuyun Li, Aijuan Zhang, Tajiguli Kupi, Yuanli Zhang, Nuerbiyemu Maimaiti, Ailimiguli Tuerhong, Xiao Hou, Lin Liu, Xiurong Zhang, Wei Geng

**Affiliations:** 1 Infection and Microbiology Research Laboratory for Women and Children, Shandong Provincial Maternal and Child Health Care Hospital Affiliated to Qingdao University 584299, Jinan, China; 2 Department of Gynecology, Shandong Provincial Maternal and Child Health Care Hospital Affiliated to Qingdao University 584299, Jinan, China; 3 Department of Traditional Chinese Medicine Gynecology, Kashi Prefecture Hospital of Traditional Chinese Medicine (Prefectural Hospital of Traditional Chinese Medicine), Kashi, China; 4 Diagnosis and Treatment Center for Pelvic Floor Disease, Shandong Provincial Maternal and Child Health Care Hospital Affiliated to Qingdao University 584299, Jinan, China; 5 Department of Traditional Chinese Medicine, Shandong Provincial Maternal and Child Health Care Hospital Affiliated to Qingdao University 584299, Jinan, China; Debreceni Egyetem, Debrecen, Hungary

**Keywords:** dehydronuciferine, fluconazole, *Candida albicans*, antifungal synergy, drug resistance

## Abstract

The increasing resistance of *Candida albicans* to conventional antifungal agents poses a major clinical challenge, underscoring the urgent need for novel therapeutic strategies. Drug combination therapy has emerged as a promising approach to overcome antifungal resistance. In this study, we evaluated the synergistic antifungal activity of dehydronuciferine (DHN), a natural aporphine alkaloid, in combination with fluconazole (FLC) against six clinically drug-resistant *C. albicans* isolates. Strong synergism was confirmed by checkerboard microdilution assay, with fractional inhibitory concentration index (FICI) values ranging from 0.063 to 0.265. Notably, for strain CA10, the minimum inhibitory concentration of FLC was reduced from >512 to 0.25 μg/mL in the presence of DHN (FICI = 0.125), representing a >2,000-fold reversal of resistance. In a *Galleria mellonella* infection model, the DHN + FLC combination significantly improved larval survival compared with either monotherapy (*P* < 0.01). Phenotypic assays demonstrated that the combination markedly suppressed key virulence traits, including hyphal growth, biofilm formation, and epithelial adhesion. Transcriptomic profiling revealed that these phenotypic changes were associated with the downregulation of hypha- and adhesion-related genes and the ergosterol biosynthesis pathway. Together, these findings identify dehydronuciferine as a promising antifungal chemosensitizer that enhances fluconazole efficacy against drug-resistant *C. albicans* through multitarget perturbation of resistance- and virulence-associated processes. Further mechanistic and preclinical studies are warranted to evaluate its translational potential.

**IMPORTANCE:**

Drug-resistant *Candida albicans* infections are increasingly difficult to treat, and the pipeline for new antifungal drugs remains limited. In this study, we show that the natural aporphine alkaloid dehydronuciferine (DHN) acts synergistically with fluconazole to potently inhibit drug-resistant *C. albicans* by simultaneously suppressing hyphal growth, biofilm formation, and epithelial adhesion. In a *Galleria mellonella* infection model, the DHN–fluconazole combination significantly improved survival compared with either agent alone. This combination strategy, which pairs an approved antifungal with a natural compound, offers a promising approach to combat antifungal resistance and may accelerate the development of alternative therapeutic options without the need for entirely new drug classes.

## INTRODUCTION

Invasive fungal infections (IFIs) pose a major public health challenge and are associated with up to 3.75 million deaths annually worldwide (1, 2). The COVID-19 pandemic has further exacerbated the incidence and mortality of IFIs (3). Invasive candidiasis is the most common hospital-associated invasive fungal infection and is characterized by high incidence, high mortality, and substantial treatment costs (1, 4). *Candida albicans* is the most frequently isolated *Candida* species, accounting for around 40%–80% of *Candida* isolates recovered from patients in hospitals (5). Clinically, *C. albicans* is responsible for a broad spectrum of infections, ranging from mucosal colonization to life-threatening bloodstream infections, with candidemia incidence reaching up to 52% and mortality rates approaching 40% (6, 7). In the United States, *C. albicans* accounts for an estimated 60,000 cases of systemic candidiasis annually, with attributable healthcare costs ranging from US$2–4 billion (8). Accordingly, the World Health Organization has classified *C. albicans* as a “critical priority” fungal pathogen in its first Fungal Priority Pathogens List (9).

Drug-resistant fungal infections have emerged as an increasingly urgent clinical challenge, contributing substantially to global morbidity and mortality (6). Among fungal pathogens, drug-resistant *C. albicans* represents an escalating global health threat. Although *C. albicans* has historically remained susceptible to most antifungal agents, resistance has increased markedly over the past two decades, largely driven by extensive antifungal exposure in clinical settings (8). Currently, azole antifungal agents, particularly fluconazole (FLC), are the first-line treatment for *C. albicans* infections because of their broad spectrum, high efficacy, good oral bioavailability, and favorable safety profile (10). However, the long-term, widespread, and sometimes inappropriate use of FLC worldwide has intensified resistance in *C. albicans*. Fluconazole resistance in *C. albicans* is a multifactorial process rather than the consequence of a single genetic alteration. One of the best-characterized mechanisms is the overexpression of ATP-binding cassette transporters (Cdr1 and Cdr2) and the major facilitator superfamily transporter Mdr1, which actively reduce intracellular azole accumulation (11). In parallel, mutations in *ERG11*, encoding the azole target lanosterol 14α-demethylase, diminish fluconazole binding affinity (12), while altered expression of *ERG11* and other ergosterol biosynthetic genes further remodels membrane sterol composition and promotes azole resistance (11, 12). Beyond these target-related mechanisms, structured biofilms surrounded by a protective extracellular matrix confer intrinsic tolerance through restricted drug penetration, metabolic adaptation, and persister cell formation (13). Furthermore, activation of stress response pathways, particularly the molecular chaperone Hsp90, stabilizes signaling proteins involved in azole tolerance and morphogenesis, thereby facilitating the evolution and maintenance of resistant phenotypes (14). Importantly, these resistance mechanisms frequently coexist and interact, highlighting the complexity of fluconazole resistance and underscoring the need for therapeutic strategies capable of simultaneously targeting multiple resistance-associated pathways (11). Epidemiological data indicate that in some regions, the resistance rate of vaginal *C. albicans* isolates to FLC can reach 71.5% (10). Moreover, drug-resistant *C. albicans* strains can spread within populations, further complicating control efforts and representing a major cause of clinical treatment failure and recurrence (15–17). Given the limited number of highly effective and low-toxicity antifungal drugs available, drug-resistant *C. albicans* has become a pressing scientific problem that urgently needs to be addressed in antifungal therapy. Therefore, developing new therapeutics to overcome fungal resistance is an immediate and critical challenge in current antifungal drug research.

Drug combination therapy has emerged as an effective strategy for combating antimicrobial resistance and is widely used in the treatment of diseases such as malaria, tuberculosis, HIV infection, and multidrug-resistant bacterial infections (18, 19). By combining agents with distinct mechanisms of action, this approach can produce synergistic effects, reduce the required dosage of individual drugs, minimize toxicity, delay or reverse resistance development, and broaden antimicrobial spectra. In the context of fungal infections, combination strategies have attracted increasing attention (20, 21). Investigating drug combination therapies against drug-resistant *C. albicans* holds significant research value. FLC, a classic triazole antifungal, remains widely used in clinical practice due to its favorable safety profile and high oral bioavailability. Drug combination strategies can enhance the efficacy of first-line agents such as FLC by reducing the fungal burden and thereby lowering the probability of resistance emergence (19, 22). Consequently, screening for non-antifungal agents that can synergize with FLC against drug-resistant *C. albicans* is of great importance. Identifying such drug combinations could rejuvenate the clinical utility of FLC, a safe and cost-effective drug, and provide a practical strategy against drug-resistant *C. albicans*.

Natural alkaloids have attracted increasing interest as sources of antimicrobial adjuvants because of their structural diversity and broad pharmacological activities (23–25). Dehydronuciferine (DHN), an aporphine-type isoquinoline alkaloid isolated from *Nelumbo nucifera*, has been reported to exhibit anti-inflammatory, antioxidant, and antibacterial activities (26). Increasing evidence indicates that structurally related isoquinoline alkaloids, including berberine and chelerythrine, possess antifungal activity or synergize with conventional antifungal agents against drug-resistant *Candida* species (27, 28). These observations suggest that DHN may also possess previously unrecognized antifungal chemosensitizing activity. However, whether DHN exhibits antifungal activity against *C. albicans* or enhances fluconazole efficacy has not yet been investigated. Therefore, in the present study, we systematically evaluated the antifungal activity of DHN in combination with fluconazole against drug-resistant *C. albicans* using both *in vitro* and *in vivo* models. We further explored the potential mechanisms underlying the observed synergistic activity through phenotypic characterization and RNA sequencing (RNA-seq). Our findings provide experimental evidence supporting DHN as a promising antifungal chemosensitizer and establish a foundation for developing combination strategies to improve fluconazole efficacy against drug-resistant *C. albicans*.

## MATERIALS AND METHODS

### Strains and media

Six drug-resistant *C. albicans* strains obtained from Shandong Provincial Qianfoshan Hospital (Jinan, China) were used in this study. All strains were grown on yeast extract–peptone–dextrose (YPD) agar medium. Before each experiment, fungal cells were propagated in YPD medium at 35°C overnight with rotation at 200 rpm. Roswell Park Memorial Institute (RPMI) 1640 medium (Gibco) was used for biofilm formation, hyphal induction, and adhesion assays.

### Antifungal agents and *Galleria mellonella* larvae

DHN and FLC were purchased from Dalian Meilun Biotech Co., Ltd. (Dalian, China). DHN was dissolved in sterile distilled water to a stock concentration of 5,120 μg/mL, and FLC was dissolved in sterile distilled water to 2,560 μg/mL. Both stock solutions were stored at −20°C. *Galleria mellonella* larvae were purchased from Tianjin Huiyude Biotech Co., Ltd. (Tianjin, China). Larvae weighing 0.25 ± 0.03 g were used in all assays.

### Susceptibility to antifungal drugs

#### Determination of minimum inhibitory concentrations of planktonic cells *in vitro*

The antifungal activity of the combination of DHN and FLC was assessed in a suspension assay by the checkerboard method in accordance with Clinical and Laboratory Standards Institute (CLSI) guidelines for yeasts (CLSI M27-Ed4) (29). Serial twofold dilutions of the drugs were prepared in RPMI 1640. The final concentration of fungal suspension in the 96-well microplates was 1 × 10^3^ cells/mL. After adding the drugs and fungal suspension, the 96-well microplates were incubated at 35°C for 24 h, and then planktonic cell growth was observed. After incubation at 35°C for 24 h, the MIC_50_ was determined as the lowest drug concentration that inhibited ≥50% of planktonic growth compared with the drug-free control.

The fractional inhibitory concentration index (FICI) was calculated as follows: FICI = (MIC of DHN in combination / MIC of DHN alone) + (MIC of FLC in combination / MIC of FLC alone). FICI ≤0.5 was defined as synergy, 0.5 < FICI ≤ 4 as no interaction, and FICI >4 as antagonism (30, 31). All checkerboard assays were performed in triplicate (technical replicates) and independently repeated on three separate occasions (biological replicates). Minimum inhibitory concentration (MIC) values and FICIs are presented as the median of three independent experiments.

Strain CA10 was selected for subsequent assays because, among the six fluconazole-resistant *C. albicans* clinical isolates tested, it exhibited the most pronounced and reproducible synergistic reversal of resistance. In combination with DHN, the MIC of FLC for CA10 decreased from >512 to 0.25 μg/mL (FICI = 0.125), representing a >2,000-fold reduction and converting a highly resistant phenotype to a fully susceptible one, thereby providing a robust model for investigating the underlying mechanisms.

#### Disk diffusion assays

Cells from a fresh overnight culture were harvested, washed, and resuspended in phosphate-buffered saline (PBS) to 1.0 × 10^6^ cells/mL. An aliquot of 200 μL of the suspension was spread on YPD agar plates. Cellulose disks containing FLC (0.5 μg/disk), DHN (4 μg/disk), or the combination were placed on the agar surface. Plates were incubated at 35°C for 48 h and then photographed. Disk diffusion assays were performed in three independent experiments, each yielding consistent results; representative images are shown.

### Determination of antifungal effects using the *G. mellonella* infection model *in vivo*

#### Survival curve assay

*G. mellonella* survival assays were carried out as previously described (32). Larvae weighing 0.25 ± 0.03 g were employed in all assays. Strain CA10 was cultured in YPD for 24 h, washed, and resuspended in PBS. Larvae were inoculated with *C. albicans* strain CA10 suspension via the posterior proleg (5 × 10⁶ cells/larva in 10 μL PBS). At 2.5 h post-infection, infected larvae were treated with FLC (0.5 μg/larva), DHN (4 μg/larva), or the combination. An infected, PBS-treated group served as the control. Each treatment group contained 15 larvae, and the entire experiment was independently repeated three times. Larvae were kept in petri dishes at 35°C and monitored daily for survival. Death was defined as no movement after touching with metal tweezers.

#### Histopathological study

*G. mellonella* larvae were infected and treated as described above. At 2 days post-infection, one larva per group was randomly collected from each independent experiment (three independent experiments in total). Larvae were washed, fixed in 4% paraformaldehyde for 24 h, and embedded in optimal cutting temperature compound. Tissue sections (8 μm thick) were obtained using a freezing microtome and processed for periodic acid–Schiff (PAS) staining as previously described (33). The images presented are representative of each treatment group and are not intended for quantitative analysis.

### Synergistic mechanism explorations

#### Hyphal growth assay

*C. albicans* CA10 cells were activated overnight, harvested, and resuspended in RPMI 1640 medium. A 200 μL aliquot of the cell suspension (2.0 × 10⁵ cells/mL) was added to 96-well plates and treated with FLC (0.5 μg/mL), DHN (4 μg/mL), or their combination. Untreated cells served as the control. Plates were incubated at 37°C, and morphological changes were observed and photographed under an inverted microscope at 0, 3, 6, 9, and 24 h. For each condition, at least five random fields were imaged. The experiment was independently repeated three times, and representative images are shown.

#### Biofilm assays

Overnight-activated *C. albicans* CA10 cells were adjusted to 1 × 10⁶ cells/mL in RPMI 1640. A 100 μL aliquot was seeded into each well of a 96-well plate and incubated at 37°C for 24 h to allow biofilm formation. The supernatant was then carefully removed, and non-adherent cells were washed away with PBS. Fresh RPMI 1640 containing serial dilutions of FLC, DHN, or their combination was added, and the plate was incubated for another 24 h at 37°C. Untreated biofilms served as controls.

##### Crystal violet assay for biofilm biomass

After incubation, wells were washed three times with PBS, fixed with 100 μL of methanol for 15 min, and air-dried for 30 min. Biofilms were stained with 50 μL of 1% crystal violet for 5 min, washed thoroughly, and dried at 37°C for 2–3 h or overnight at room temperature. The bound dye was solubilized with 100 μL of 30% glacial acetic acid at 37°C for 30 min, and absorbance was measured at 570 nm.

##### 3-(4,5-Dimethylthiazol-2-yl)−2,5-diphenyltetrazolium bromide assay for biofilm viability

Biofilm viability was assessed using the 3-(4,5-dimethylthiazol-2-yl)−2,5-diphenyltetrazolium bromide (MTT) reduction assay. After drug treatment, the culture supernatant was removed, and wells were gently washed twice with 200 μL of sterile PBS to remove residual medium and non-adherent cells. Subsequently, 50 μL of MTT solution (1.5 mg/mL in PBS) was added to each well, and the plate was incubated at 37°C for 1.5 h in the dark. The MTT solution was then carefully aspirated, and 150 μL of dimethyl sulfoxide (DMSO) was added to dissolve the formazan crystals (30°C, 180 rpm, 10 min). Complete solubilization of formazan was confirmed visually before measurement. Absorbance was measured at 570 nm using a microplate reader. Each plate included a blank control (RPMI 1640 medium without fungal cells, processed identically) for background correction. The absorbance of the untreated biofilm (positive control, set as 100% viability) was used to normalize the data. Relative biofilm viability (%) was calculated as [(OD_570_ of treated wells − OD_570_ of blank) / (OD_570_ of untreated control − OD_570_ of blank)] × 100.

Each drug concentration was tested in triplicate wells per experiment, and all biofilm experiments were independently repeated three times (*n* = 3 biological replicates).

### Adhesion of bronchial epithelial cells

Human bronchial epithelial (16HBE) cells were cultured to confluent monolayers in 24-well plates. Cells were infected with *C. albicans* CA10 at an a multiplicity of infection (MOI) of 1:10 in RPMI 1640 and simultaneously treated with FLC (0.5 μg/mL), DHN (4 μg/mL), or the combination. After 2 h at 37°C, non-adherent fungal cells were removed by gentle PBS washing. Adherent cells were fixed with 4% paraformaldehyde and stained with Calcofluor White (CFW, 10 μg/mL). Fluorescence images were captured with a Nikon Eclipse Ti2 microscope (40× objective). Adhesion was quantified by measuring mean fluorescence intensity and the fungal adhesion area ratio (CFW-positive area of the experimental group relative to that of the control) using ImageJ. Each condition was tested in triplicate wells, and the assay was independently repeated three times.

### RNA-seq analysis

Overnight cultures of *C. albicans* CA10 were diluted to 1 × 10⁶ cells/mL in RPMI 1640 and incubated at 35°C for 6 h under four conditions, including untreated control, FLC alone (0.5 μg/mL), DHN alone (4 μg/mL), and DHN + FLC (0.5 + 4 µg/mL). Cells were collected by centrifugation and washed twice with PBS, and total RNA was extracted using a Yeast RNA Kit (Omega Bio-Tek, USA). RNA quality was assessed using NanoDrop ND-1000 and Agilent 2100 Bioanalyzer; samples with RNA integrity number (RIN) >7.0 were used for library preparation.

Library construction and sequencing were performed by Novogene Bioinformatics Technology Co., Ltd. (Beijing, China). Indexed libraries were prepared according to standard protocols, and sequencing was carried out on an Illumina HiSeq platform to generate 150 bp paired-end reads. Raw reads were filtered to remove adapters, poly-N sequences, and low-quality bases. Gene expression was quantified as fragments per kilobase per million mapped reads (FPKM). Differential expression analysis between the combination group and the control was performed using edgeR, with thresholds of |log_2_ fold change| >1 and adjusted *P* < 0.05. GO and KEGG enrichment analyses were conducted using clusterProfiler (v3.8.1) with a significance threshold of corrected *P* < 0.05. Three independent biological replicates were prepared for each of the four conditions.

### Statistics analysis

All experiments were independently repeated three times. Data are presented as mean ± standard deviation. Normality and homoscedasticity were assessed using the Shapiro–Wilk test and Levene’s test, respectively. For comparisons among multiple groups meeting parametric assumptions, one-way analysis of variance (ANOVA) followed by Bonferroni post hoc test was applied; when assumptions were violated, the non-parametric Kruskal–Wallis test with the Dunn post hoc test was used. For datasets with a 2 × 2 factorial design, two-way ANOVA was performed to evaluate main effects and interactions. Survival curves were analyzed by the Kaplan–Meier method and compared with the log-rank test. All statistical tests were two-tailed, and a *P* < 0.05 was considered statistically significant (*P < 0.05, **P < 0.01, ***P < 0.001). Statistical analyses and graph generation were performed using GraphPad Prism (v7.3.0; GraphPad, La Jolla, CA, USA) and SPSS (v22; IBM, Armonk, NY, USA).

## RESULTS

### DHN combination with FLC exhibits synergistic antifungal activity against planktonic drug-resistant *C. albicans in vitro*

**Fig 1:**
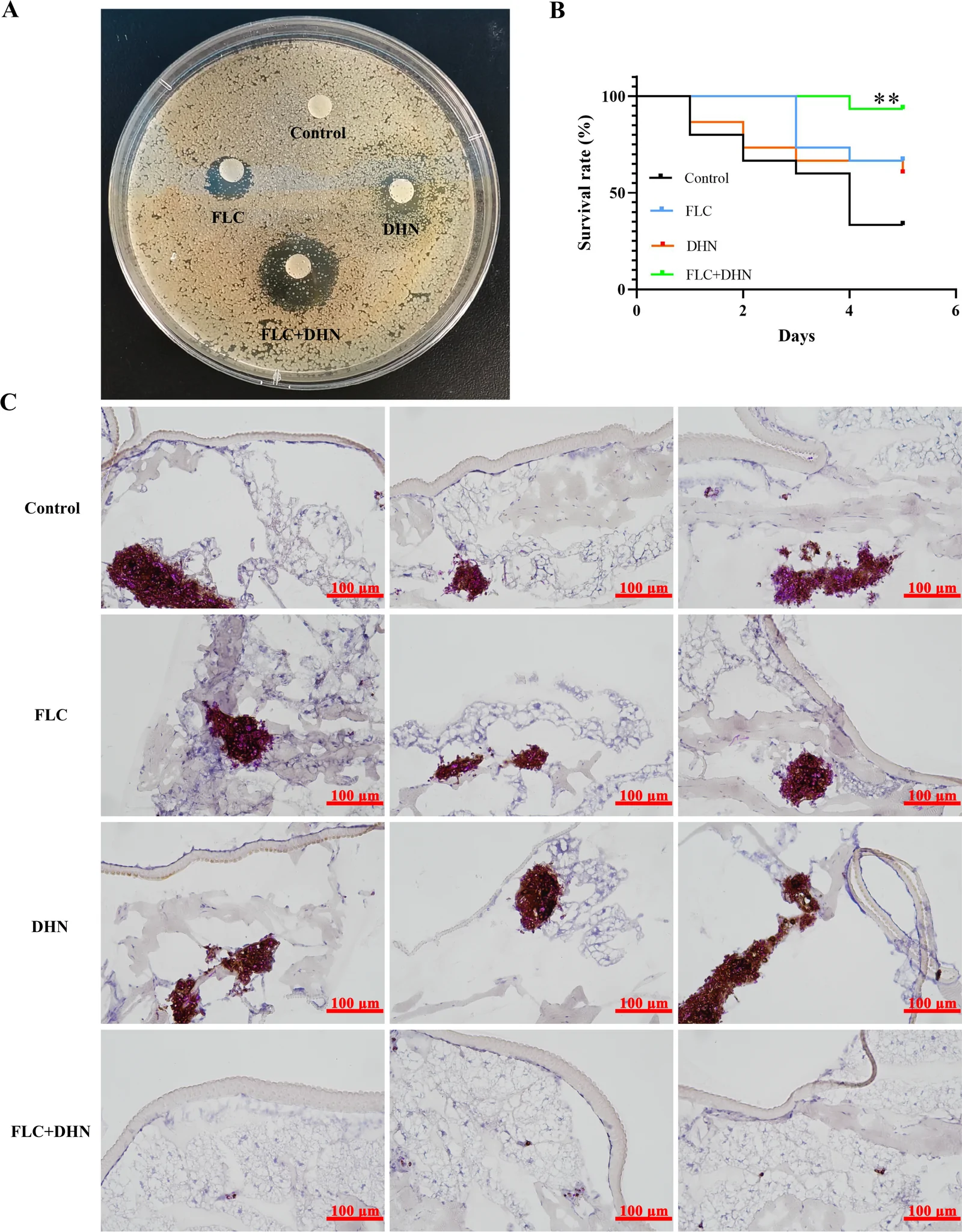
Synergistic antifungal effects of DHN combined with FLC against drug-resistant *C. albicans* strain CA10 *in vitro* and *in vivo*. (**A**) Agar disk diffusion assay of DHN + FLC against CA10. CA10 (2 × 10⁵ CFU) was spread on YPDA plates, and cellulose disks impregnated with FLC (0.5 µg/mL) and/or DHN (4 µg/mL) were placed on the agar surface. The plates were incubated at 30°C for 48 h prior to the observation of inhibitory effects. The image is representative of three independent experiments. (**B and C**) DHN combined with FLC enhanced survival rate and reduced fungal burden in the CA10-infected *G. mellonella* model. (**B**) Survival curves. Larvae were inoculated via the posterior proleg with approximately 2 × 10^5^ cells of drug-resistant *C. albicans* strain CA10. After 2 h of infection, the larvae were treated with PBS, DHN (4 μg/larva), FLC (0.5 μg/larva), or DHN + FLC (4 + 0.5 µg/larva). Survival was monitored daily for 5 days. Data are from one experiment with 15 larvae per group (*n* = 15). Survival curves were compared using the Kaplan–Meier method with the log-rank test (**P < 0.01). (**C**) Histopathological sections. Larvae were sacrificed 2 days post-infection, and tissues were processed for PAS staining. Representative images from three independent experiments are shown. Scale bar = 40 µm. All presented histopathological micrographs are representative images from each group, rather than results from a quantitative or statistically supported analysis.

To evaluate whether DHN and FLC act synergistically against drug-resistant *C. albicans*, we first evaluated their combination *in vitro* using the checkerboard assay according to CLSI M27-Ed4 (29). DHN alone exhibited limited antifungal activity, whereas the DHN–FLC combination showed strong synergistic effects across the tested concentration ranges (DHN, 0.25–2 μg/mL; FLC, 0.25–8 μg/mL), with FICI values ranging from 0.063 to 0.265 (Table 1). According to established criteria, synergy was defined as FICI ≤0.5. Consistent with the checkerboard results, disk diffusion assays showed that DHN or FLC alone produced only weak inhibition zones, whereas the combination generated a markedly larger inhibition zone against strain CA10 (Fig. 1A). Together, these results indicate that DHN markedly enhances the antifungal activity of FLC against planktonic drug-resistant *C. albicans in vitro*.

**TABLE 1:** Effect of DHN combined with FLC against drug-resistant *C. albicans in vitro^a^^,^*^*b*^

Antifungal agents	Strains	MIC (μg/mL)				FICI model	
		MIC_FLC_	*C*_FLC_	MIC_DHN_	*C*_DHN_	FICI	IN
DHN + FLC	CA10	>512	0.25	16	2	0.125	SYN
	CA16	>512	0.25	2	0.5	0.250	SYN
	CA103	>512	0.5	16	1	0.063	SYN
	CA137	>512	8	1	0.25	0.265	SYN
	CA632	>512	8	1	0.25	0.265	SYN
	CA20003	>512	2	16	1	0.066	SYN

^*a*^ DHN, dehydronuciferine; FLC, fluconazole; *C*_DHN_, concentration of DHN at the synergistic combination point; *C*_FLC_, concentration of FLC at the synergistic combination point; MIC, minimum inhibitory concentration of a single drug that inhibits fungal growth by 50% compared with the drug-free control; FICI, fractional inhibitory concentration index; IN, interpretation; SYN, synergism.

^*b*^ MIC values and FICIs are shown as the median of three independent experiments.

### DHN combined with FLC improves therapeutic efficacy in a *Galleria mellonella* infection model

Having established synergistic activity *in vitro*, we next evaluated the therapeutic efficacy of the DHN–FLC combination *in vivo* using a *Galleria mellonella* infection model. Larvae were infected with a lethal inoculum of *C. albicans* strain CA10 and subsequently treated with DHN, FLC, or the combination. Neither FLC nor DHN alone significantly improved survival compared with the control, whereas the combination group exhibited a markedly higher survival rate, particularly on days 4 and 5 post-treatment (*P* < 0.01, Fig. 1B). To qualitatively assess fungal invasion, representative larval specimens from each treatment group were collected 2 days post-infection and subjected to histopathological examination (33). Representative tissue sections from the untreated control showed extensive PAS-positive fungal structures, indicating successful fungal invasion. In contrast, sections from the combination-treated group exhibited visibly fewer fungal structures than those from the control and monotherapy groups (Fig. 1C). These observations are consistent with the improved survival of larvae receiving the combination treatment. Collectively, these findings indicate that the DHN–FLC combination improves therapeutic efficacy against drug-resistant *C. albicans* in the *G. mellonella* infection model.

### DHN combined with FLC suppresses virulence-associated phenotypes of drug-resistant *C. albicans*

**Fig 2:**
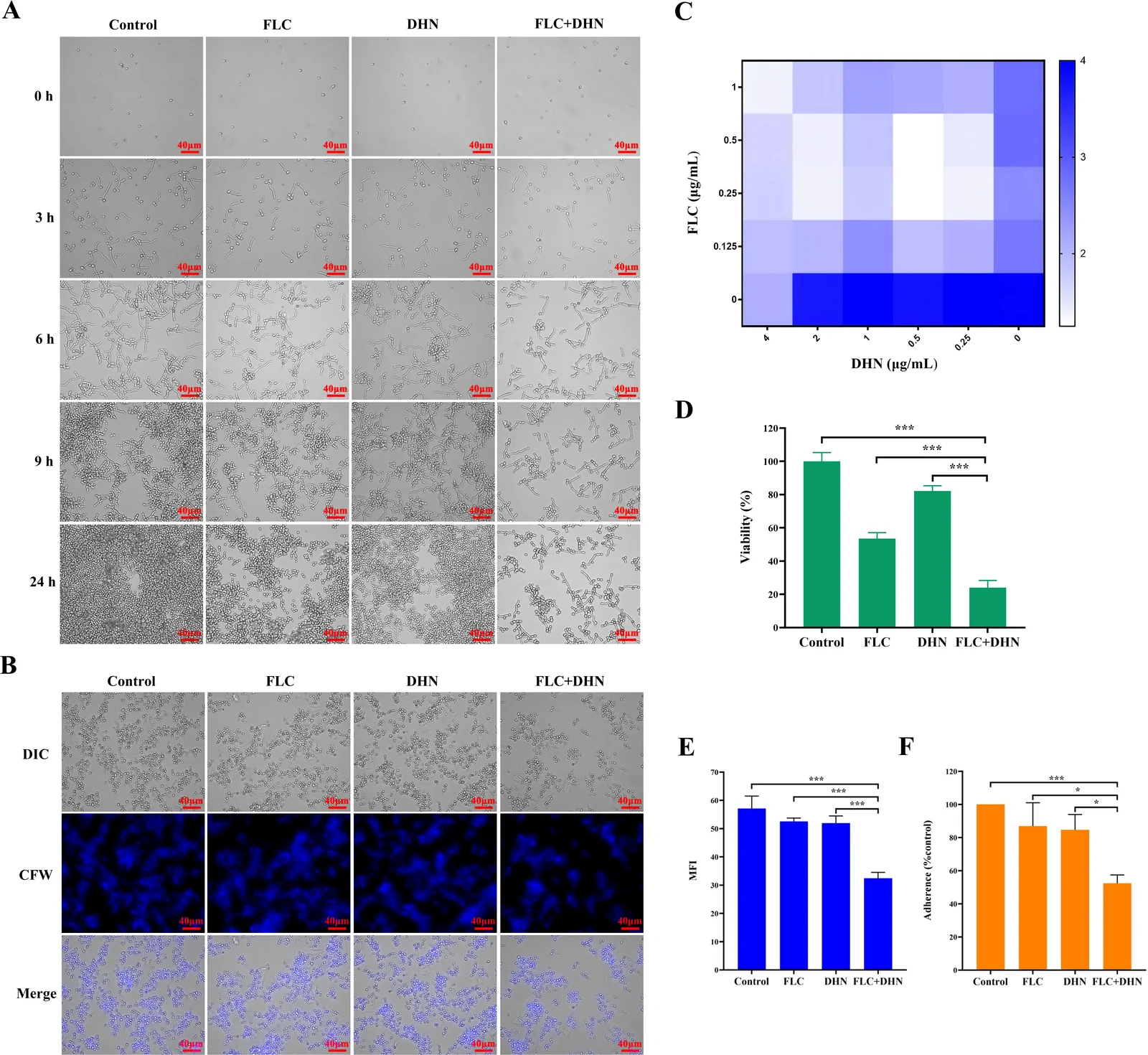
DHN in combination with FLC inhibits hyphal growth, biofilm formation, and adhesion to host epithelial cells in the drug-resistant *C. albicans* strain CA10. (**A**) DHN combined with FLC inhibited hyphal growth. CA10 cells (2 × 10⁵ cells/mL) were treated with DHN (4 µg/mL) and/or FLC (0.5 µg/mL) in RPMI 1640. Morphology was photographed at 0, 3, 6, 9, and 24 h. Images are representative of three independent experiments. (**B and C**) DHN combined with FLC inhibited biofilm formation, as assessed by crystal violet staining (**B**) and the MTT reduction assay (**C**). (**B**) Biofilms of CA10 in 96-well plates were treated with serial drug concentrations for 24 h, then stained with crystal violet. (**C**) Biofilm metabolic activity was assessed by the MTT reduction assay following treatment with DHN (4 µg/mL), FLC (0.5 µg/mL), or their combination. Relative viability (%) is shown with the untreated control set as 100%. Values represent the mean ± standard deviation (SD) of three independent biological replicates (*n* = 3). (**D–F**) DHN combined with FLC inhibited CA10 adhesion to bronchial epithelial cells. (**D**) Fluorescence microscopy of CFW-stained CA10 adhering to HBE monolayers after 2 h under control, FLC (0.5 μg/mL), DHN (4 μg/mL), or combination. Scale bar: 40 μm. Images are representative of three independent experiments. (**E and F**) Quantitative analysis of adhesion: mean fluorescence intensity (**E**) and fungal adhesion area ratio (**F**). Data are presented as mean ± SD of three independent biological replicates (*n* = 3). In each independent experiment, each condition was tested in triplicate wells (technical replicates). Statistical comparisons were performed using the values from the three independent biological experiments as the experimental units. Normality and homoscedasticity were confirmed by Shapiro–Wilk and Levene tests, respectively. Groups were compared by one-way ANOVA followed by the Bonferroni post hoc test. *P < 0.05, ***P < 0.001.

The pathogenicity of *C. albicans* is intimately associated with its capacity for hyphal morphogenesis, biofilm formation, and adhesion to host surfaces, traits that also contribute to antifungal tolerance (4, 34). We therefore investigated whether the DHN–FLC combination could attenuate these virulence-associated phenotypes. Hyphal morphogenesis was assessed by incubating strain CA10 in RPMI 1640 medium at 37°C for 24 h in the presence of DHN, FLC, or the combination. Untreated cells developed abundant elongated hyphae, whereas the combination treatment produced markedly fewer and shorter hyphae than either monotherapy (Fig. 2A). Biofilm formation was evaluated by crystal violet staining (Fig. 2B) and MTT reduction assay (Fig. 2C). The combination caused a dose-dependent reduction in biofilm biomass as measured by crystal violet, while at the synergistic concentration (DHN 4 µg/mL + FLC 0.5 µg/mL), it significantly reduced biofilm metabolic activity compared with the untreated control and single-drug treatments (Fig. 2B and C). Adhesion to host epithelial cells was evaluated using 16HBE cells. Fluorescence microscopy demonstrated extensive fungal attachment in the untreated control group, whereas combination treatment markedly reduced fungal adhesion compared with either single agent alone (Fig. 2D). Quantitative analysis confirmed that both the mean fluorescence intensity and fungal adhesion area ratio were significantly lower in the combination group than in the control, DHN-alone, or FLC-alone groups (Fig. 2E and F). Taken together, these results indicate that DHN enhances the antifungal activity of FLC in association with reduced hyphal development, biofilm formation, and epithelial adhesion in drug-resistant *C. albicans*.

### Transcriptomic analysis identifies pathways associated with the synergistic activity of DHN and FLC

**Fig 3:**
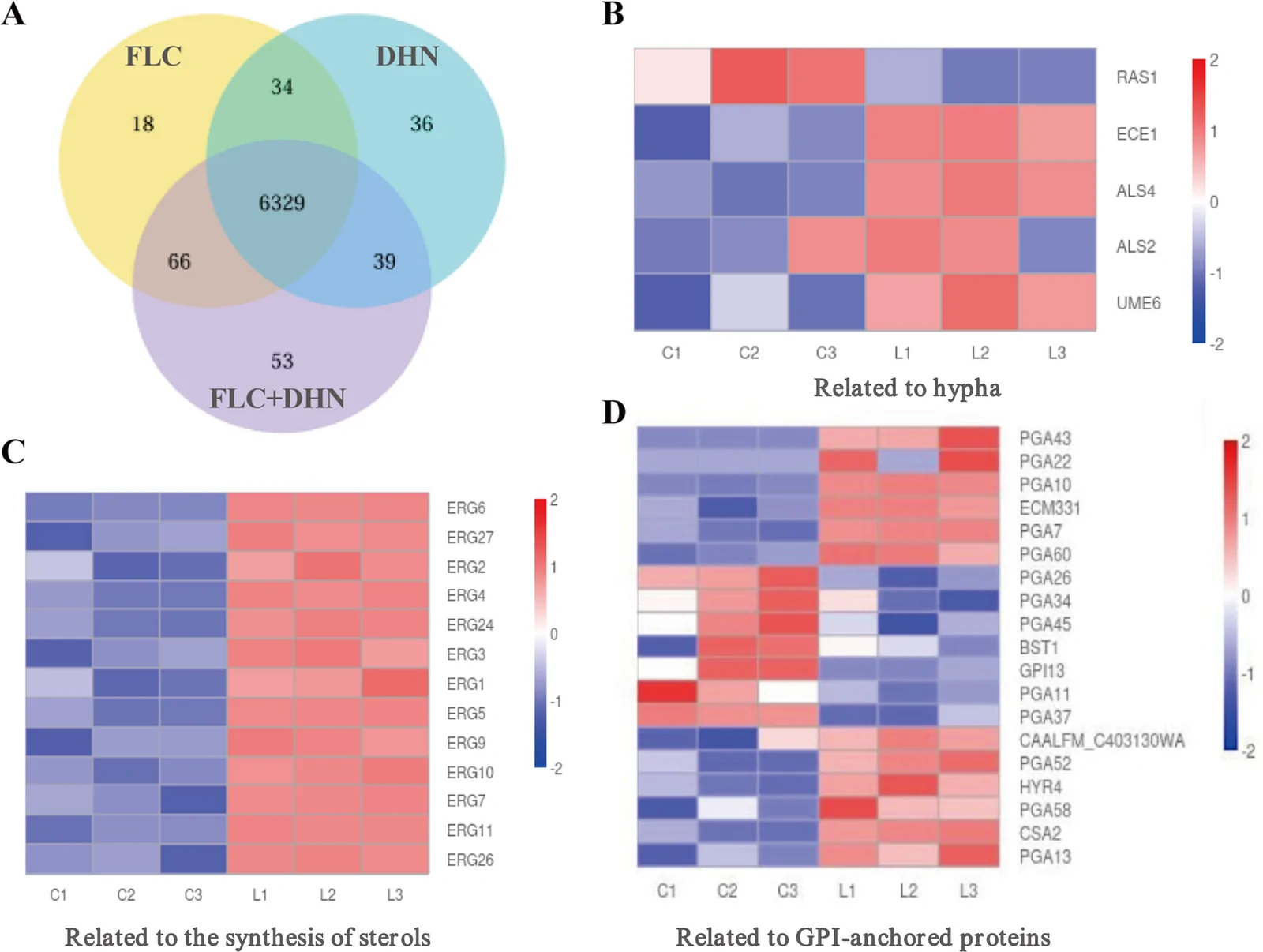
RNA-Seq analysis of drug-resistant *C. albicans* treated by DHN combined with FLC. (**A**) Venn diagram of differentially expressed genes (|log_2_ fold change| > 1, adjusted *P* < 0.05) between the DHN + FLC combination group and the untreated control group. (**B–D**) Heatmaps of the main upregulated and downregulated genes in the drug combination. Gene expression levels with log_2_(FPKM) values are represented by colors, with red and blue corresponding to high and low expression, respectively. C1–C3 represent three biological replicates of the untreated control group, while L1–L3 represent three biological replicates of the DHN + FLC group (*n* = 3 per group). Differentially expressed genes were identified using DESeq2.

To further explore the transcriptional responses associated with the synergistic activity of DHN and FLC in drug-resistant *C. albicans*, RNA-seq was performed. The combination treatment produced a distinct gene expression profile compared with the control group (Fig. 3A). Differentially expressed genes were mainly enriched in three functional categories, including hyphal morphogenesis (e.g., *ALS* family genes, *ECE1*, and *UME6*), biosynthesis of GPI-anchored cell wall proteins (e.g., *PGA* family genes), and ergosterol biosynthesis (e.g., *ERG* family genes) (Fig. 3B through D). Notably, the downregulation of hypha-related genes such as *ALS3*, *HWP1*, and *ECE1* was consistent with the reduced hyphal development and biofilm formation observed phenotypically. Likewise, altered expression of GPI-anchored cell wall protein genes was consistent with the reduced epithelial adhesion detected following combination treatment. In addition, the transcriptomic analysis identified altered expression of multiple ergosterol biosynthesis-related genes, suggesting potential changes in membrane organization that may contribute to enhanced fluconazole susceptibility. Overall, these transcriptomic alterations are associated with the synergistic antifungal activity of DHN combined with FLC against drug-resistant *C. albicans* and identify candidate biological pathways for future functional validation.

## DISCUSSION

*C. albicans* remains a leading cause of invasive fungal infections worldwide, posing formidable clinical and economic challenges (6). The clinical management of these infections is further complicated by the fact that fluconazole resistance in *C. albicans* is driven by multiple complementary mechanisms, including drug efflux, alterations in ergosterol biosynthesis, biofilm-associated tolerance, and stress-response pathways (6, 35). Consequently, combination therapy has emerged as an attractive strategy for restoring azole susceptibility while minimizing the emergence of additional resistance (36, 37). Combining conventional azoles with non-antifungal natural products has gained increasing attention as a promising strategy to restore the activity of compromised antifungal agents (38). In the present study, we demonstrate for the first time that the natural aporphine alkaloid DHN markedly potentiates the antifungal activity of fluconazole against drug-resistant *C. albicans*. The strong synergistic activity was observed *in vitro* and was further supported by improved survival in the *G. mellonella* infection model, suggesting that the *in vitro* interaction translates into biologically meaningful therapeutic efficacy *in vivo*. Although the *G. mellonella* model provides valuable preliminary evidence of *in vivo* efficacy, it cannot substitute for mammalian toxicity or pharmacokinetic evaluation. Future studies should therefore include mammalian cell cytotoxicity assays together with systemic efficacy and safety assessments in mammalian infection models.

Phenotypic analyses indicated that the enhanced antifungal activity of the DHN–FLC combination was associated with reduced epithelial adhesion, impaired hyphal morphogenesis, and diminished biofilm formation, all of which are critical determinants of *C. albicans* colonization, persistence, and pathogenicity (39–41). Because biofilms substantially limit antifungal penetration and harbor highly tolerant fungal subpopulations, simultaneous inhibition of adhesion, filamentation, and biofilm development is likely to improve fluconazole accessibility and therapeutic efficacy (42). In our study, the combination markedly restricted hyphal elongation, which likely weakened biofilm architecture and may have improved FLC accessibility to fungal cells. While the present hyphal inhibition data are based on representative micrographs and qualitative morphological assessment, future studies incorporating quantitative metrics, such as hyphal length measurements, or a filamentation index—will be valuable to further substantiate these observations and strengthen the mechanistic conclusions.

Transcriptomic analysis further showed that combination treatment was associated with coordinated alterations in gene networks involved in hyphal morphogenesis, GPI-anchored cell wall protein biosynthesis, and ergosterol metabolism. The reduced expression of *ALS3*, *HWP*1, and *ECE1* was consistent with the observed inhibition of filamentation and biofilm formation (43), whereas altered expression of GPI-anchored adhesin genes corresponded with reduced epithelial attachment (44, 45). In addition, differential expression of ergosterol biosynthesis genes suggests potential alterations in membrane organization that may contribute to enhanced fluconazole susceptibility (46). As RNA sequencing identifies transcriptional associations, candidate pathways and causal mechanisms require further functional validation. Future studies combining targeted genetic manipulation with transcriptomic and biochemical analyses will help clarify the molecular basis of the observed synergistic interaction. Another limitation of the present study is that the genetic background of strain CA10 and the additional clinical *C. albicans* isolates remain to be characterized. Whole-genome sequencing and characterization of resistance-associated determinants, including *ERG11*, *TAC1*, *MRR1*, and *UPC2*, will facilitate interpretation of strain-specific responses and provide further insight into the mechanisms underlying fluconazole sensitization.

DHN belongs to the aporphine alkaloid family, a structurally distinct subclass of benzylisoquinoline alkaloids that has recently attracted increasing attention because of its diverse pharmacological activities. Although antifungal investigations of aporphine alkaloids remain relatively limited, emerging evidence suggests that members of this family possess promising antifungal potential. For example, glaucine, another naturally occurring aporphine alkaloid, has recently been identified through subtractive proteomics and molecular docking analyses as a potential anti-*Candida auris* compound. Mechanistically, glaucine was predicted to competitively bind fungal glycerol-3-phosphate O-acyltransferase, thereby disrupting membrane lipid biosynthesis while also weakly inhibiting mevalonate kinase to interfere with ergosterol production (47). Compared with glaucine, DHN exhibited a broader antifungal potentiating profile by restoring fluconazole susceptibility while simultaneously suppressing adhesion, hyphal morphogenesis, biofilm formation, and the transcription of virulence- and ergosterol-associated genes. These findings suggest that DHN may represent a multifunctional antifungal chemosensitizer within the aporphine alkaloid family. Nevertheless, whether these activities are common to aporphine alkaloids or reflect structural features unique to the dehydrogenated scaffold of DHN remains to be clarified through systematic structure–activity relationship studies. Our findings are also consistent with previous reports showing that natural alkaloids can function as antifungal chemosensitizers. Chelerythrine has been shown to synergize with fluconazole by suppressing planktonic growth, early biofilm development, and hyphal morphogenesis in drug-resistant *C. albicans* (28), whereas berberine enhances azole susceptibility primarily through inhibition of biofilm formation (27). Unlike these previously reported alkaloids, DHN was associated with broader phenotypic and transcriptional alterations, suggesting that its fluconazole-potentiating activity may involve coordinated modulation of multiple resistance-related pathways rather than a single dominant target. To our knowledge, this is the first study demonstrating that an aporphine alkaloid restores fluconazole susceptibility in drug-resistant *C. albicans* while simultaneously characterizing the associated transcriptional response and virulence-related phenotypes.

**Fig 4:**
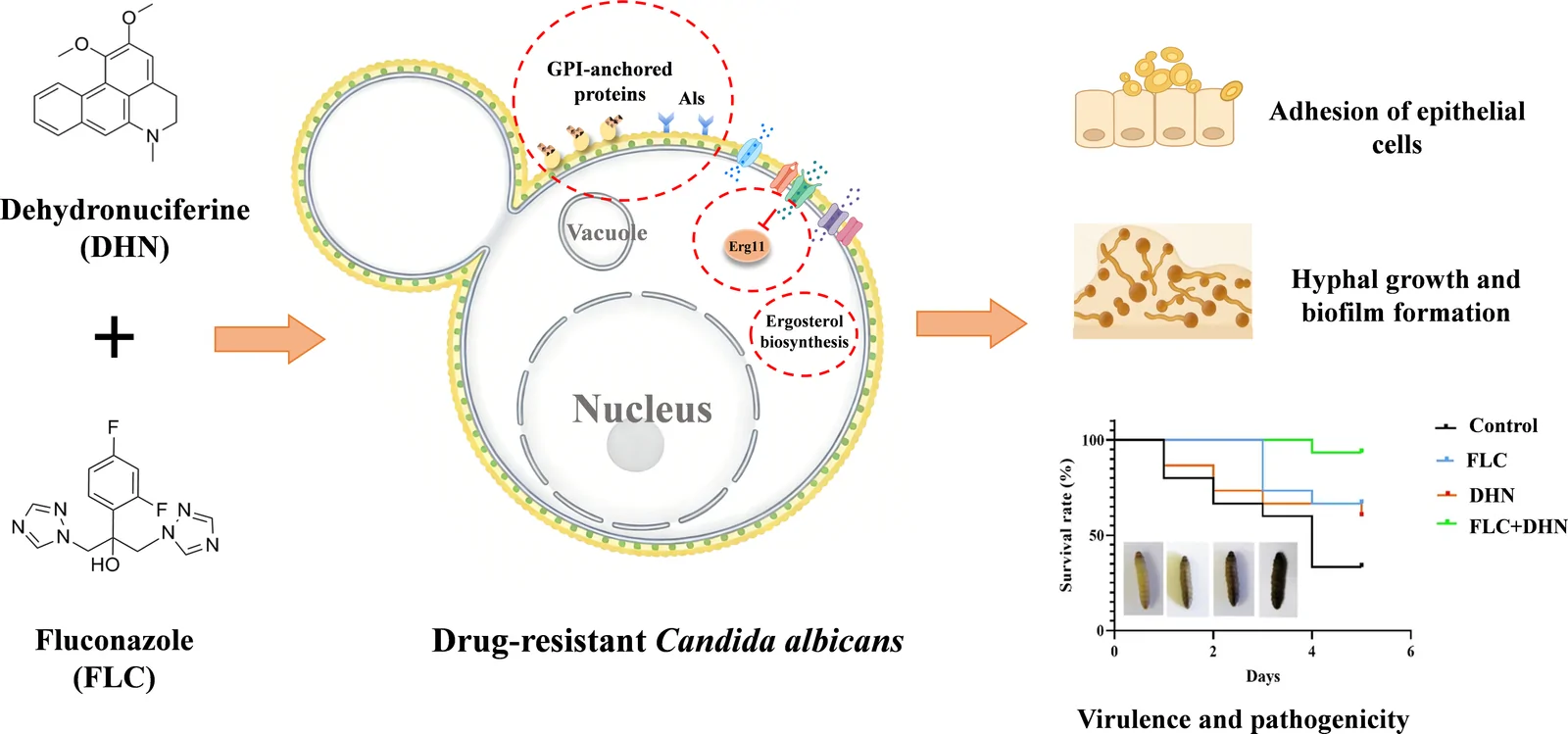
Schematic diagram illustrating the synergistic antifungal mechanisms of DHN combined with FLC against drug-resistant *C. albicans*. The combination of DHN and FLC enhances antifungal activity against drug-resistant *C. albicans* by transcriptionally downregulating genes involved in GPI-anchored cell wall protein biosynthesis and ergosterol metabolism while simultaneously suppressing hyphal growth, disrupting biofilm architecture, and reducing epithelial adhesion. In addition, alterations in surface-associated molecular features may modulate host–*C*. *albicans* interactions and contribute to improved fungal clearance. See text for details.

Collectively, our phenotypic findings and transcriptomic profiling data support a proposed working model in which DHN enhances fluconazole activity against drug-resistant *C. albicans* by concurrently weakening a spectrum of resistance-associated cellular processes, including fungal adhesion, filamentation, biofilm development, and membrane-related physiological functions (Fig. 4). Adhesion to host or abiotic surfaces is the initiating step in biofilm formation, and this process is driven largely by GPI-anchored adhesins such as *ALS* family proteins (41). Subsequent hyphal morphogenesis further provides the structural scaffold for mature three-dimensional biofilm architecture and promotes the expression of hypha-associated factors that support extracellular matrix production and biofilm persistence (39, 40). Given that mature *C. albicans* biofilms are well recognized to confer intrinsic tolerance to azoles and other antifungal agents (48, 49), the marked inhibition of adhesion, hyphal elongation, and biofilm biomass observed in our study is likely to contribute substantially to the restored fluconazole susceptibility in drug-resistant strains. In addition, the transcriptional suppression of genes implicated in ergosterol biosynthesis and GPI-anchored cell wall protein biogenesis is consistent with the hypothesis that DHN may disrupt fungal membrane organization and lipid-raft-dependent processes, both of which are tightly associated with azole resistance and fungal virulence (50, 51). Although the precise molecular mechanisms underlying this synergistic effect require further validation through targeted functional assays, these findings establish a mechanistic framework for subsequent investigations and support the development of DHN as a promising antifungal chemosensitizing agent.

### Conclusion

In summary, this study demonstrates that the natural aporphine alkaloid DHN acts synergistically with fluconazole against drug-resistant *C. albicans* both *in vitro* and in the *Galleria mellonella* infection model. The enhanced antifungal activity was associated with inhibition of hyphal morphogenesis, biofilm formation, and epithelial adhesion, together with transcriptional alterations in pathways related to cell wall organization and ergosterol biosynthesis. These findings identify DHN as a promising antifungal chemosensitizing candidate with the potential to improve fluconazole efficacy against drug-resistant *C. albicans*. Future studies should prioritize mammalian cytotoxicity and pharmacokinetic evaluations, together with targeted functional assays to validate the proposed mechanisms, as essential steps toward assessing the translational potential of this combination strategy.

## Data Availability

Data supporting the findings of this study are available from the corresponding author upon reasonable request.
